# Food Preparation Skills, Digital Diet Literacy, Mediterranean Diet Adherence, and Ultra-Processed Food Consumption Among Adults Residing in Istanbul: A Cross-Sectional Study

**DOI:** 10.3390/nu18162585

**Published:** 2026-08-07

**Authors:** Hatice Merve Bayram, Arda Ozturkcan

**Affiliations:** Department of Nutrition and Dietetics, Faculty of Health Sciences, Istanbul Gelisim University, 34310 Istanbul, Turkey; turkcana@hotmail.com

**Keywords:** ultra-processed foods, food skills, cooking skills, digital diet literacy, Mediterranean diet, dietary behavior

## Abstract

**Background:** Ultra-processed food (UPF) consumption may be associated not only with nutrition knowledge but also with individuals’ practical food-related competencies and overall dietary patterns. **Aim:** This study investigated how cooking and food skills, dietary literacy, and adherence to the Mediterranean diet were related to UPF consumption among adults in İstanbul, Türkiye. **Methods:** This descriptive cross-sectional study included 293 (85.7% female, mean age of 22.63 ± 4.66 years) adults residing in Istanbul, Türkiye between 1 January 2026 and 30 June 2026. Data were collected through an online questionnaire comprising sociodemographic and food-related characteristics, the Cooking Skills and Food Skills Measure, the e-Healthy Diet Literacy Scale (e-HDL), the Mediterranean Diet Adherence Screener (MEDAS), and the Short Screening Questionnaire of Highly Processed Food Consumption (sQ-HPF). Validated Turkish versions of all instruments were used; no measurement tool was developed specifically for this study. **Results:** 57.0% of participants were classified as having a high level of UPF consumption. The high level of UPF consumption group was younger, cooked at home less frequently, ordered food more frequently, and had lower cooking skills, food skills, and MEDAS scores compared to the low level of UPF consumption group (all *p* < 0.05). UPF consumption was negatively correlated with food skills (r: −0.33), MEDAS (r = −0.28), cooking skills (r = −0.27), age (r = −0.21), and home-cooking frequency (r = −0.18) and positively correlated with food-ordering frequency (r = 0.18). In the final regression model, younger age (β = −0.144, *p* = 0.009), lower food skills (β = −0.325, *p* < 0.001), and lower MEDAS scores (β = −0.252, *p* < 0.001) were independently associated with higher UPF consumption. E-HDL was not independently associated with UPF consumption. The final model explained 18.4% of the adjusted variance. **Conclusions:** Among this predominantly young and female sample of adults residing in Istanbul, lower food skills and lower Mediterranean diet adherence co-occurred with higher UPF consumption. Although the direction of these associations cannot be determined, the findings may inform future strategies that integrate nutrition education with practical food preparation support and the promotion of Mediterranean-style food choices.

## 1. Introduction

Ultra-processed foods (UPFs) are defined within the NOVA food classification system by Monteiro et al. as “formulations of ingredients, mostly of exclusive industrial use, that result from a series of industrial processes” [1]. This category includes numerous ready-to-consume products, such as packaged snack foods, soft drinks, instant noodles, and pre-prepared meals [1]. UPFs represent a significant and growing portion of the global food supply, although their contribution to dietary energy varies considerably across countries [2,3]. A systematic review of worldwide consumption data showed that UPFs generally accounted for more than 50% of total energy intake in the United States and the United Kingdom, whereas lower contributions were reported in several Mediterranean and middle-income countries [4].

From 2007 to 2022, UPF sales increased across low- and middle-income countries while remaining high in many high-income countries [3]. In Türkiye, available nationally derived data indicate that UPFs account for 30.64% of total daily energy intake, placing their contribution below that reported in the United States and the United Kingdom but demonstrating that they constitute a substantial component of the national diet [5,6]. The principal sources of UPF-derived energy in Türkiye were ultra-processed breads, fats, and pastries [6]. Nevertheless, evidence on UPF consumption in Türkiye remains limited, and no comprehensive national strategy specifically addressing UPFs has been implemented. However, data on the sale and consumption of UPF is quite limited, and there is no policy or strategy in place nationwide. According to the Türkiye Nutrition and Health Survey, the average daily energy intake in Türkiye was 1912.56  ±  736.36 kcal, of which 605.30  ±  422.69 kcal—corresponding to 30.64%—was derived from UPFs [6]. The most consumed UPF groups were ultra-processed breads (61.2%), non-alcoholic beverages (16.1%), and pastries (6.3%). The energy contribution of UPFs was ranked as ultra-processed breads (62.87%), fats (9.9%), and pastries (8.7%) [6]. Istanbul, as Türkiye’s largest and highly urbanized metropolitan area, provides a particularly relevant setting for examining UPF consumption. In this setting, time pressure, commuting demands, the widespread availability of packaged convenience products, and increasing access to food-delivery services and digital platforms may increase the importance of convenience in food selection. Nevertheless, findings obtained from adults residing in Istanbul should not be considered representative of the broader adult population of Türkiye, particularly populations living in rural areas or cities with different socioeconomic and food-environment characteristics.

This increase in UPF product sales has been observed to contribute to energy intake [1,7]. UPFs typically have an unfavorable nutritional profile, characterized by high levels of energy, saturated fat, refined carbohydrates, free sugars, and sodium, alongside relatively low amounts of fiber, protein, and essential micronutrients [1,8]. Therefore, the increased consumption of UPFs also has a negative impact on health and is associated with obesity [9], type 2 diabetes [10], cardiovascular diseases [11], cancer [12], and an increase in the risk of all-cause mortality [13,14].

UPFs are inexpensive and their preparation often involves simple processes [15]. By reducing the time and effort required for food preparation, UPFs are particularly well suited to the demands of fast-paced modern lifestyles [15,16]. Longer working hours, limited discretionary time, and extended commuting periods may discourage home cooking and increase dependence on UPF [17]. Moreover, reduced interest in food preparation, the gradual weakening of cooking-related competencies, and the declining routine use of these skills may further promote UPF consumption [1,18,19]. However, UPF consumption is shaped not only by nutrition knowledge and food skills but also by socioeconomic, demographic, household, and environmental conditions. Factors such as income, education, employment status, household structure, sex, age, time availability, and product accessibility may affect individuals’ resources and opportunities for purchasing and preparing food [2,15,16]. These factors may therefore confound the associations between food-related competencies and UPF consumption and should be considered, where available, in adjusted analyses.

In parallel with changes in the food supply, the digital information environment surrounding food choices has also expanded substantially [20]. Nutritional information is widely disseminated in digital environments, with adults increasingly accessing it through social media, websites, mobile apps, influencers, and online food marketing. However, while these platforms may improve access to nutrition information, they may also expose individuals to misinformation, persuasive advertising and the promotion of highly palatable UPFs [20,21]. Therefore, nutrition literacy plays a crucial role in enabling individuals to evaluate nutrition information, make healthier food choices, counter misinformation, and apply practical skills when purchasing and preparing foods [16,22].

Overall diet quality also appears to be associated with UPF intake. Greater adherence to the Mediterranean diet, which emphasizes minimally processed plant foods, has been repeatedly associated with lower UPF consumption [23,24]. Thus, adherence to a high-quality traditional dietary pattern may represent an additional behavioral factor related to UPF intake, beyond cooking skills and dietary literacy.

Given their convenience, widespread availability, long shelf life, and high palatability [25], it is important to better understand the factors that influence consumers’ preference for UPFs. In the literature, it was observed that there is a relationship between cooking-related competencies, food literacy, healthy dietary patterns, and UPF consumption [16,26,27,28], but these factors have frequently been examined separately. Therefore, this study investigated whether cooking and food skills, dietary literacy, and adherence to the Mediterranean diet were associated with UPF consumption among adults in Istanbul, Türkiye.

## 2. Methods

### 2.1. Study Design and Participants

This descriptive cross-sectional study included adults living in Istanbul, Türkiye, between 1 January 2026 and 30 June 2026. Participants were recruited using non-probability convenience and snowball sampling. Data were collected using an online, self-administered questionnaire that included items on sociodemographic characteristics, the Cooking Skills and Food Skills Measure, the e-Healthy Diet Literacy Scale, the Mediterranean Diet Adherence Screener, and a short screening questionnaire for UPF consumption (sQ-HPF). The online survey link was initially distributed through social media platforms and was subsequently shared by participants within their networks. Because the survey link was openly circulated, the number of individuals who viewed or received the invitation could not be determined, and therefore a response rate could not be calculated.

The sample size was estimated a priori using G*Power version 3.1 for a two-tailed bivariate correlation analysis. An effect size of r = 0.20 was selected to permit the detection of weak but potentially meaningful associations between food-related competencies and UPF consumption. This conservative choice was preferred to a medium effect size because behavioral and dietary outcomes are typically influenced by multiple interacting factors, and stronger associations were not assumed. With α = 0.05 and 80% power, the analysis indicated that approximately 199 participants were required. The final sample of 293 participants exceeded this minimum requirement.

Participants were eligible if they were aged 18 years or older, resided in Istanbul, were able to read and understand Turkish, provided informed consent, and completed the online questionnaire. Eligible respondents were excluded from the analytical dataset if they were pregnant or lactating, had been diagnosed with cancer, had followed a specific therapeutic or restrictive diet within the previous six months, or submitted incomplete, internally inconsistent, duplicate, or anthropometrically implausible data. Other chronic diseases were not considered exclusion criteria; their presence was recorded as a participant characteristic.

A total of 300 individuals completed the questionnaire. Seven participants were excluded from the analysis: two because they reported following a restrictive diet, one because of pregnancy, two because of missing anthropometric data, one because of an incomplete questionnaire, and one because of an implausible response (e.g., reporting a body weight of 200 kg). The final sample comprised 293 participants. The sample was predominantly female (86.7%) and relatively young (mean age: 22.63 ± 4.66 years); 18.4% reported having a chronic disease. Consequently, the sample should not be considered representative of the adult population of Istanbul. The online, non-probability recruitment procedure may have introduced self-selection and coverage bias by favoring younger individuals, women, social media users, and those with greater interest in nutrition-related topics.

### 2.2. Ethical Statement

The Istanbul Gelisim University Ethics Committee approved this study, with the number 2025-21 and date of 1 December 2025, and the principles of the Declaration of Helsinki were followed. Before accessing the questionnaire, participants were provided with electronic information about the study and were required to indicate their informed consent by selecting the relevant confirmation option. Individuals who did not provide electronic informed consent were not permitted to proceed with the questionnaire.

### 2.3. Sociodemographic and Anthropometric Characteristics

Sociodemographic and lifestyle information, including age, sex, chronic disease status, regular exercise, sleep patterns, social media use, home-cooking frequency, and food-ordering frequency, was collected using self-reported questionnaire items. Age was recorded in completed years. Body weight (kg) and height (cm) were self-reported by the participants. Body mass index (BMI) was calculated as body weight in kilograms divided by height in meters squared (kg/m^2^).

### 2.4. Cooking Skills and Food Skills Measure

The Cooking Skills and Food Skills Measure was developed by Lavelle et al. [29] to assess individuals’ knowledge, skills, and self-efficacy related to cooking and food skills. Its Turkish adaptation, including validity and reliability, was studied by Keleş and Ok [30]. The measure comprises two separately scored subscales: Cooking Skills (14 items; possible score range: 0–98) and Food Skills (19 items; possible score range: 0–133). For each item, participants who performed the relevant skill rated how well they performed it on a scale ranging from 1 (“very poor”) to 7 (“very good”). The instrument also includes a single combined response option, “never/rarely perform,” which is scored as 0. This option and its scoring were retained from the validated Turkish version and were not created or combined specifically for the present study. Item scores were summed separately for each subscale, with higher scores indicating greater perceived cooking or food skills. The Cronbach’s alpha value for this study was found to be 0.983.

### 2.5. E-Healthy Diet Literacy Scale

The e-Healthy Diet Literacy Scale (e-HDL), originally developed by van Duong et al. [31], incorporates four items adapted from the Digital Healthy Diet Literacy scale. It includes 15 items grouped into five domains: digital healthy diet literacy and the ability to access, understand, evaluate, and apply electronic information on healthy eating. The Turkish version was validated by Onbaşı and Türker [32]. Items in the first domain are rated on a five-point frequency scale ranging from 1 (“never”) to 5 (“every day”), with intermediate options representing “a few times a year,” “a few times a month,” and “a few times a week.” In the second dimension, each correct response is scored as 5, whereas incorrect responses and “I do not know” responses are scored as 1. For the third dimension, responses are scored from 1 (“strongly disagree”) to 5 (“strongly agree”). For the fourth dimension, responses are scored as 1 for “never,” 2 for “rarely,” 3 for “sometimes,” 4 for “often,” and 5 for “always.” For the fifth dimension, responses are scored as 1 for “very difficult,” 2 for “somewhat difficult,” 3 for “somewhat easy,” and 4 for “very easy.” The total scale score is calculated by summing the scores for all items. Higher scores indicate a higher level of electronic healthy diet literacy. The Cronbach’s alpha value for this study was found to be 0.761.

### 2.6. Mediterranean Diet Adherence Screener

Schröder et al. [33], and Martínez-González et al. [34] developed the Mediterranean Dietary Adherence Screener (MEDAS). Pehlivanoğlu et al. [35] established the Turkish validity and reliability of the scale. The scale consists of 14 questions, each scoring 0 or 1 point based on consumption amounts, and the total score is calculated from this. Based on previously published interpretations, scores below 7 were classified as low adherence, scores of 7–8 as moderate adherence, and scores of 9 or higher as high adherence [35]. These thresholds were not developed specifically for the present study. The Cronbach’s alpha value for this study was found to be 0.752.

### 2.7. Short Screening Questionnaire of Highly Processed Food Consumption

UPF consumption was evaluated using the Short Screening Questionnaire for Highly Processed Food Consumption (sQ-HPF), originally developed by Martinez-Perez et al. [36] and subsequently adapted and validated in Turkish by Erdoğan Gövez et al. [37]. Despite the use of the term “highly processed foods” in its title, the instrument assesses foods that correspond conceptually to the ultra-processed food category within the NOVA framework. The questionnaire includes 11 items covering different UPF groups, with each item assigned a score of 0 or 1 according to reported consumption frequency. Total sQ-HPF scores range from 0 to 11. In accordance with the cutoff proposed for the original instrument and retained in its Turkish validation, scores of ≥6 were classified as high UPF consumption [36,37]. No study-specific cutoff was developed. The Cronbach’s alpha value for this study was found to be 0.828.

### 2.8. Statistical Analysis

All analyses were conducted using SPSS version 24.0. Descriptive statistics were calculated for each variable, with continuous data reported as mean ± standard deviation and categorical data as frequencies and percentages. Data distributions were examined using the Kolmogorov–Smirnov test. Because the continuous variables were not normally distributed, comparisons between participants classified as having low and high UPF consumption according to the sQ-HPF cutoff were performed using the Mann–Whitney U test. The variables compared between these two groups were age, BMI, home-cooking frequency, food-ordering frequency, cooking skills, food skills, e-HDL, and MEDAS scores. The Kruskal–Wallis H test was used to compare these variables across the low, moderate, and high Mediterranean-diet-adherence groups defined by the MEDAS score. When a significant overall difference was identified, Dunn–Bonferroni-adjusted post hoc tests were used for pairwise comparisons. Spearman’s rank correlation analysis was used to examine associations among age, BMI, home-cooking frequency, food-ordering frequency, and cooking skills, food skills, e-HDL, MEDAS, and sQ-HPF scores. Hierarchical multiple linear regression analysis was conducted to determine the predictors of UPF consumption, with predictors entered in six sequential blocks. In the hierarchical regression analysis, age, sex, and BMI were entered in the first block as covariates. These variables were selected a priori based on previously reported associations with UPF consumption and dietary behavior, as well as their availability in the dataset, rather than on the basis of statistical significance in the present sample. Age and sex were included as demographic covariates, whereas BMI was included as an anthropometric covariate. Education, income, employment status, and household composition were not collected and therefore could not be included in the model. Multicollinearity was assessed using tolerance and variance inflation factor (VIF) values, and the independence of residuals was checked with the Durbin–Watson statistic. The significance threshold for all tests was set at *p* < 0.05.

## 3. Results

Table 1 shows the demographic characteristics of participants. A total of 293 participants (85.7% female; the mean age was 22.63 ± 4.66 years, and the mean BMI was 23.12 ± 3.95 kg/m^2^) completed the study. Most participants (81.6%) reported no chronic disease, while 64.8% did not exercise regularly and 54.6% had irregular sleep patterns. All participants reported using social media, and 46.8% used it for more than 60 min per day. Participants cooked at home an average of 3.64 ± 2.40 days per week and ordered food online 2.15 ± 1.83 times per week.

Table 2 presents the comparison of variables according to the MEDAS classification. The cooking skills total score differed significantly across the MEDAS adherence groups (*p*: 0.030). Pairwise comparisons indicated significant differences between the low- and high-adherence groups and between the moderate- and high-adherence groups. Similarly, sQ-HPF scores differed significantly among all MEDAS adherence groups (*p* < 0.001).

Table 3 presents the comparison of some variables according to sQ-HPF classification. The high level of UPF consumption group was younger than those with low UPF consumption (*p*: 0.002) and cooked at home less frequently (*p*: 0.049). Food-ordering frequency also differed significantly between the groups (*p*: 0.005), although the median frequency was 2.00 times/week in both groups. The high level of UPF consumption group had a lower cooking skills score (*p* < 0.001), food skills score (*p* < 0.001), and MEDAS score (*p* < 0.001).

Table 4 shows the correlation between age, BMI, cooking frequency, food-order frequency, and the scales. There was a moderate negative correlation between home-cooking frequency and food-order frequency (r: −0.33, *p* < 0.001) and a weak positive correlation between the cooking skills (r: 0.21, *p* < 0.001), food skills (r: 0.17, *p* < 0.001), e-HDL (r: 0.12, *p* < 0.05), and MEDAS scores (r: 0.12, *p* < 0.05). Food-order frequency was weak and negatively correlated with cooking skills and food skills scores (both r: −0.24, *p* < 0.001) and e-HDL score (r: −0.14, *p* < 0.05). There was a moderate negative correlation between the sQ-HPF total score and food skills score (r = −0.33, *p* < 0.001), while there was a weak negative correlation between the sQ-HPF total score and cooking skills score (r: −0.27, *p* < 0.001), MEDAS score (r: −0.28, *p* < 0.001), age (r: −0.21, *p* < 0.001), and home-cooking frequency (r: −0.18, *p* < 0.001). Additionally, the sQ-HPF total score showed a weak positive correlation with food-order frequency (r: 0.18, *p* < 0.001).

Table 5 presents the hierarchical regression analysis examining factors associated with UPF consumption. Model 1, which included sociodemographic characteristics, explained 5.6% of the variance in UPF consumption. Adding food-related behaviors in Model 2 significantly increased the explained variance by 3.0% (*p* = 0.009). Cooking skills and food skills provided additional contributions of 3.6% in Model 3 (*p* < 0.001) and 2.6% in Model 4 (*p* = 0.004), respectively. The addition of Mediterranean diet adherence in Model 5 produced the largest improvement in model fit (*ΔR*^2^ = 0.060, *p* < 0.001), increasing the total explained variance to 20.9%. In contrast, adding e-HDL in Model 6 did not improve the model (*ΔR*^2^ < 0.001, *p* = 0.675). In the final model, younger age (*β* = −0.144, *p* = 0.009), lower food skills (*β* = −0.325, *p* < 0.001), and lower MEDAS scores (*β* = −0.252, *p* < 0.001) were independently associated with higher UPF consumption. VIF values ranged from 1.03 to 1.48 across the predictors, indicating no evidence of problematic multicollinearity. The final model explained 18.4% of the adjusted variance.

## 4. Discussion

This study contributes exploratory evidence examining practical food-related competencies, digital healthy diet literacy, dietary patterns, and UPF consumption within the same analytical framework. Within this predominantly young and female convenience sample from Istanbul, practical food management competencies and overall dietary patterns appeared to be more informative correlates of UPF consumption than food-related behaviors or access to digital dietary information alone. This distinction is relevant because possessing nutrition-related information may not necessarily translate into the practical ability to select, purchase, and prepare minimally processed foods. However, these patterns are specific to the characteristics and recruitment context of the present sample and should not be interpreted as population-level estimates for adults in Istanbul or Türkiye.

The findings should also be interpreted within the Turkish food environment. National data indicate that ultra-processed breads constitute the most frequently consumed UPF category and the largest source of UPF-derived energy in Türkiye, followed by products such as non-alcoholic beverages, pastries, and industrial fats [6]. Thus, UPF consumption in Türkiye may not be limited to products commonly perceived as fast food or ready meals but may also involve routinely consumed packaged breads, bakery products, sweetened beverages, biscuits, confectionery, and packaged snack foods. This product profile may differ from that of countries in which ready meals, breakfast cereals, or processed meat products make larger contributions to UPF intake. Moreover, the wide availability of packaged bakery and convenience products in Istanbul may partly shape the relationship between food skills and UPF consumption. The use of a broad category-based screening instrument should also be considered when interpreting these findings within the Turkish food environment. Although the sQ-HPF captures the commonly consumed categories of highly processed foods, it may not fully represent locally specific products, preparation practices, or differences in processing within broad food categories. This issue may be particularly relevant in Türkiye, where packaged breads and bakery products constitute major sources of UPF-derived energy and where products that appear similar may differ in formulation and degree of processing. Consequently, the sQ-HPF score reflects the frequency of consumption of selected food categories rather than the full complexity or quantitative contribution of UPFs to the diet.

UPF consumption is increasing, especially in late adolescence and young adulthood. In the present study, younger age was independently associated with higher UPF consumption, consistent with previous findings [4,16,38,39,40]. Among young adults, food choices may be shaped by higher energy needs, social influences, limited nutrition knowledge, and constraints related to the time, resources, or equipment required for purchasing and preparing healthy meals. These factors may increase their preference for UPFs, which are convenient, readily available, and highly palatable [16,41,42]. Accordingly, educational initiatives and intervention strategies designed to encourage healthier dietary choices among young adults, with the aim of reducing UPF consumption, are of considerable importance [43]. By fostering the development of sustainable healthy eating behaviors, these approaches may enable young people to make more informed food choices in the long term. However, the participants in this study were predominantly young adults, and the relatively narrow age distribution limits the extent to which this finding can be generalized across the adult life course.

Food and cooking skills constitute a multidimensional concept that incorporates one’s level of knowledge, confidence, and attitudinal orientation toward this area. However, this subject remains relatively scarce in the literature [16,19,44]. Lam and Adams [19] reported that better home food skills and more frequent use of these skills were generally associated with lower UPF consumption. Mengi Çelik et al. [16] found inverse associations between UPF consumption and both cooking and food skills among Turkish adults. In the present study, food skills, but not cooking skills, remained independently associated with UPF consumption. Cooking skills primarily reflect confidence and competence in performing cooking-related tasks, whereas food skills encompass broader competencies related to meal planning, food selection, resource management, and the organization of food preparation. Although conceptually distinct, the two constructs were strongly correlated. The VIF values did not indicate problematic multicollinearity; nevertheless, their shared variance may have reduced the unique contribution of cooking skills in the mutually adjusted model. Therefore, the absence of an independent association for cooking skills should not be interpreted as evidence that cooking skills are unimportant. Within the limits of the cross-sectional design, the findings indicate that broader food management competencies and UPF consumption co-occurred more consistently than technical cooking ability and UPF consumption. However, the direction of this relationship cannot be established. Lower food skills may increase reliance on convenient ultra-processed products, whereas frequent reliance on UPFs may also reduce opportunities or motivation to plan meals, select ingredients, and develop food preparation skills. The concept of “food agency” similarly emphasizes that dietary practices arise from the interaction of technical skills, cognitive capacities, available resources, and social and environmental conditions rather than cooking competence in isolation [45].

Home cooking is an important component to tackle poor quality diets. There is increasing evidence of an association between poor quality diet, away-from-home food consumption [26], and the consumption of UPF [19,46]. Among US adults, cooking dinner at home more frequently and spending more time cooking were associated with greater consumption of unprocessed or minimally processed foods and lower consumption of UPF [46]. Lam et al. [19] reported that individuals who cooked a main meal at least five days a week consumed a lower percentage of dietary energy from UPFs [19]. A Turkish study showed that lower frequency of cooking was associated with higher UPF consumption [16]. Although home-cooking and food-ordering frequencies were associated with UPF consumption in the unadjusted analyses, neither remained independently associated with UPF consumption in the final model. This attenuation may indicate that frequency-based measures do not fully capture how food-related behaviors and UPF consumption are related; however, the cross-sectional data do not allow the temporal direction of these relationships to be determined. Cooking at home can still involve ready-made sauces, packaged meals, processed meats, and other UPFs, whereas food ordered outside the home is not necessarily UPF. Accordingly, frequency-based indicators may not adequately capture the processing level or nutritional quality of the foods consumed.

The Mediterranean diet is among the most extensively investigated dietary patterns in the scientific literature. Beyond substantial epidemiological and clinical evidence supporting its protective health effects [47], a growing number of Mediterranean countries have shifted away from this traditional eating pattern [24]. It was observed that there was an inverse association between adherence to a Mediterranean diet and UPF consumption [24,48]. We found that participants with better adherence to a Mediterranean diet have lower sQ-HPF scores, and significant differences were observed across all Mediterranean diet adherence categories. This could be explained as a nutritional transition from fresh meals and dishes that are part of the Mediterranean diet towards a higher intake of ready-to-consume and hyper-palatable food and beverage products. The intake of some foods known to be the basis of the Mediterranean diet is affected by high UPF consumption [24]. High consumption of UPFs was associated with lower fruit, vegetable, fish, and nut intake and higher cereal, fat, meat and sweetener intake [48]. Accordingly, greater adherence to the Mediterranean diet may co-occur with lower UPF consumption because Mediterranean-style dietary patterns emphasize minimally processed plant-based foods, whereas frequent UPF consumption may, conversely, displace foods characteristic of this dietary pattern. The temporal direction of this relationship cannot be determined from the present cross-sectional data. Nevertheless, Mediterranean diet adherence and UPF consumption should not be regarded as exact opposites, as they represent related but distinct dimensions of dietary quality: one reflects adherence to a traditional food-based pattern, whereas the other reflects the degree of industrial food processing.

Nutrition literacy interventions can help individuals and communities recognize the characteristics and potential health risks of UPFs, critically evaluate nutrition misinformation, and acquire practical skills for healthier food purchasing and preparation [16]. It was reported that the lowest e-HDL scores were associated with a higher consumption of UPFs; however, after adjustment, this association was not significant among Turkish Nutrition and Dietetics students [20]. Another study found that higher e-HDL scores were significantly associated with increased adherence to a MD [28]. In the present study, e-HDL was not independently associated with UPF consumption and did not improve the explanatory power of the final regression model. However, this finding should not be interpreted as evidence that digital healthy diet literacy has no role in food-related practices. The predominantly young sample recruited through online platforms may have been relatively digitally connected, potentially restricting the variability in e-HDL and reducing the ability to detect an independent association with UPF consumption. Moreover, e-HDL was positively correlated with cooking skills, food skills, and home-cooking frequency and negatively correlated with food-ordering frequency. These associations suggest that e-HDL may be related to UPF consumption indirectly through food-related competencies and behaviors rather than through a direct independent association.

The study has some limitations. First, the cross-sectional design precludes causal or temporal interpretations. Second, participants were recruited through convenience and snowball sampling using online platforms, and the sample was predominantly young and female. This recruitment approach may have introduced self-selection and coverage bias by favoring social media users and individuals with greater interest in nutrition-related topics. Therefore, the findings cannot be considered representative of adults in Istanbul or generalized to the broader Turkish adult population, particularly men, older adults, individuals with limited digital access, and populations living in rural areas or different socioeconomic and food environments. The observed associations and their potential public health implications should consequently be regarded as exploratory and context-specific. Future studies should employ probability-based or stratified sampling across regions and demographic groups to determine whether these associations persist in more representative populations. Third, all variables were self-reported and may be affected by recall and social desirability biases. Fourth, potentially relevant determinants such as income, employment and student status, living arrangements, time constraints, food insecurity, nutrition education, and exposure to digital food marketing were not included in the model.

## 5. Conclusions

The present findings identify practical food competencies and Mediterranean diet adherence as relevant correlates of UPF consumption among predominantly young and female adults in Istanbul. However, the cross-sectional design precludes determining the direction of these relationships. Lower practical food competencies may be associated with greater reliance on UPFs, while frequent UPF consumption may conversely reduce opportunities or motivation to plan and prepare meals. Population-based longitudinal and intervention studies should therefore evaluate whether integrating food preparation and meal-planning components into nutrition education can reduce UPF consumption beyond the provision of nutrition information alone. Such research should consider the local food environment and commonly consumed UPFs, including packaged breads, bakery products, sweetened beverages, and snack foods, particularly among young adults whose food practices may be shaped by convenience, time constraints, and digital food environments.

## Figures and Tables

**Table 1:** Sociodemographic, anthropometric, health, and lifestyle characteristics of the participants (*n*: 293).

Variable	*n* (%) or Mean ± SD
Sociodemographic characteristics	
Age (years) *	22.63 ± 4.66
Sex	
Male	42 (14.3)
Female	251 (85.7)
Anthropometric characteristics	
Height (cm) *	165.73 ± 8.46
Body weight (kg) *	63.89 ± 14.16
BMI (kg/m^2^) *	23.12 ± 3.95
Health and lifestyle characteristics	
Presence of chronic disease (Yes)	54 (18.4)
Regular exercise (Yes)	103 (35.2)
Regular sleep (Yes)	133 (45.4)
Social media use (Yes)	293 (100.0)
Social media duration (per day)	
<15 min	22 (7.5)
15–30 min	50 (17.1)
30–60 min	84 (28.7)
>60 min	137 (46.8)
Home cooking (days/week)	3.64 ± 2.40
Online orders/week	2.15 ± 1.83

* Data are presented as mean ± standard deviation; all other variables are presented as *n* (%). BMI, body mass index.

**Table 2:** Comparison of age, BMI, cooking and food skills, e-HDL total score, and sQ-HPF score according to the MEDAS classification.

Variable	Low Adherence (*n*: 162)	Moderate Adherence (*n:* 107)	High Adherence (*n:* 24)	*p*-Value
Age (years)	21.00 (3.00)	22.00 (3.00)	22.00 (4.25)	0.326
BMI (kg/m^2^)	22.77 (5.72)	22.94 (4.85)	21.02 (4.29)	0.496
Home-cooking frequency (days/week)	3.00 (3.75)	4.00 (3.00)	5.00 (5.00)	0.292
Food-ordering frequency (times/week)	2.00 (2.00)	2.00 (2.00)	2.00 (1.00)	0.639
Cooking Skills score ^b,c^	57.50 (39.50)	61.00 (43.50)	73.00 (30.00)	0.030 *
Food Skills score	81.00 (64.00)	81.00 (63.50)	102.50 (59.75)	0.330
e-HDL total score	38.00 (11.75)	41.00 (14.00)	42.50 (14.25)	0.149
sQ-HPF total score ^a,b,c^	6.00 (4.00)	6.00 (5.00)	4.00 (4.00)	<0.001 **

Data are presented as median (interquartile range). * *p* < 0.05, ** *p* < 0.001. The Kruskal–Wallis H test with Dunn–Bonferroni-adjusted post hoc pairwise comparisons were used. ^a^ Differences between low adherence and moderate adherence. ^b^ Differences between low adherence and high adherence. ^c^ Differences between moderate adherence and high adherence. BMI: Body mass index, e-HDL: e-Healthy Diet Literacy Scale, MEDAS: Mediterranean Diet Adherence Screener, sQ-HPF: Short screening questionnaire of highly processed food consumption.

**Table 3:** Comparison of age, BMI, cooking and food skills, and e-HDL and MEDAS total scores according to the sQ-HPF classification.

Variable	Low Level of UPF Consumption (*n*: 126)	High Level of UPF Consumption (*n*: 167)	*p*-Value
Age (years)	22.00 (3.00)	21.00 (3.00)	0.002 *
BMI (kg/m^2^)	22.54 (4.46)	22.94 (5.71)	0.545
Home-cooking frequency (days/week)	5.00 (4.00)	3.00 (3.50)	0.049 *
Food-order frequency (times/week)	2.00 (1.00)	2.00 (2.00)	0.005 *
Cooking Skills score	68.00 (32.00)	55.50 (41.75)	<0.001 **
Food Skills score	97.00 (63.00)	73.00 (57.50)	<0.001 **
e-HDL total score	41.00 (13.00)	39.00 (13.00)	0.148
MEDAS total score	7.00 (2.00)	6.00 (2.00)	<0.001 **

Data are presented as median (interquartile range). * *p* < 0.05, ** *p* < 0.001. The Mann–Whitney U test was used. UPF: ultra-processed food, e-HDL: e-Healthy Diet Literacy Scale, MEDAS: Mediterranean Diet Adherence Screener.

**Table 4:** Spearman correlation between age, BMI, cooking and food-order frequency, cooking and food skills, e-HDL, MEDAS, and sQ-HPF total scores.

Variable	1	2	3	4	5	6	7	8
1. Age	—							
2. BMI	0.17 **	—						
3. Cooking frequency days/wk	0.04	0.01	—					
4. Food-order frequency/wk	−0.10	−0.02	−0.33 **	—				
5. Cooking Skills score	0.13 *	−0.06	0.21 **	−0.24 **	—			
6. Food Skills score	0.13 *	−0.05	0.17 **	−0.24 **	0.84 **	—		
7. e-HDL total score	0.27 **	−0.01	0.12 *	−0.14 *	0.24 **	0.21 **	—	
8. MEDAS total score	0.11	−0.02	0.12 *	−0.05	0.13 *	0.10	0.10	—
9. sQ-HPF total score	−0.21 **	0.03	−0.18 **	0.18 **	−0.27 **	−0.33 **	−0.08	−0.28 **

* *p* < 0.05, ** *p* < 0.001. BMI: Body mass index, e-HDL: e-Healthy Diet Literacy Scale, MEDAS: Mediterranean Diet Adherence Screener, sQ-HPF: Short screening questionnaire of highly processed food consumption.

**Table 5:** Hierarchical multiple regression examining factors associated with UPF consumption.

Predictor	*B*	SE	95% CI for *B*	*β*	*t*	*p*
***Model 1. Sociodemographic characteristics***						
*Constant*	9.214	1.351	6.555 to 11.873	–	6.82	<0.001 **
Age	−0.147	0.040	−0.226 to −0.068	−0.211	−3.64	<0.001 **
Sex (female)	1.014	0.560	−0.088 to 2.116	0.110	1.81	0.071
BMI	−0.004	0.050	−0.102 to 0.094	−0.005	−0.08	0.935
*R^2^ = 0.056; adjusted R*^2^ *= 0.046; F(3, 289) = 5.74, p < 0.001; ΔR*^2^ *= 0.056, ΔF(3, 289) = 5.74, p < 0.001*						
***Model 2. + Food-related behaviors***						
*Constant*	8.989	1.400	6.233 to 11.745	–	6.42	<0.001 **
Age	−0.133	0.040	−0.212 to −0.054	−0.190	−3.30	0.001 *
Sex (female)	0.565	0.573	−0.563 to 1.693	0.061	0.98	0.326
BMI	0.008	0.050	−0.090 to 0.106	0.009	0.16	0.876
Home-cooking frequency (days/week)	−0.179	0.081	−0.338 to −0.020	−0.132	−2.21	0.028 *
Food-ordering frequency (times/week)	0.161	0.107	−0.050 to 0.372	0.091	1.50	0.136
*R*^2^ *= 0.087; adjusted R*^2^ *= 0.071; F(5, 287) = 5.45, p < 0.001; ΔR*^2^ *= 0.030, ΔF(2, 287) = 4.78, p = 0.009*						
***Model 3. + Cooking skills***						
*Constant*	10.573	1.449	7.721 to 13.425	–	7.30	<0.001 **
Age	−0.118	0.040	−0.197 to −0.039	−0.169	−2.97	0.003 *
Sex (female)	0.337	0.567	−0.779 to 1.453	0.036	0.59	0.553
BMI	−0.005	0.049	−0.101 to 0.091	−0.006	−0.10	0.923
Home-cooking frequency (days/week)	−0.142	0.080	−0.299 to 0.015	−0.105	−1.76	0.079
Food-ordering frequency (times/week)	0.100	0.107	−0.111 to 0.311	0.056	0.94	0.350
Cooking Skills score	−0.027	0.008	−0.043 to −0.011	−0.203	−3.45	<0.001 **
*R*^2^ *= 0.123; adjusted R*^2^ *= 0.105; F(6, 286) = 6.69, p < 0.001; ΔR*^2^ *= 0.036, ΔF(1, 286) = 11.89, p < 0.001*						
***Model 4. + Food skills***						
*Constant*	10.644	1.430	7.829 to 13.459	–	7.44	<0.001 **
Age	−0.114	0.039	−0.191 to −0.037	−0.163	−2.89	0.004 *
Sex (female)	0.317	0.559	−0.783 to 1.417	0.034	0.57	0.571
BMI	−0.005	0.049	−0.101 to 0.091	−0.006	−0.10	0.923
Home-cooking frequency (days/week)	−0.151	0.079	−0.306 to 0.004	−0.112	−1.90	0.058
Food-ordering frequency (times/week)	0.088	0.105	−0.119 to 0.295	0.050	0.84	0.402
Cooking Skills score	0.006	0.013	−0.020 to 0.032	0.043	0.42	0.677
Food Skills score	−0.026	0.009	−0.044 to −0.008	−0.295	−2.93	0.004 *
*R*^2^ *= 0.149; adjusted R*^2^ *= 0.128; F(7, 285) = 7.12, p < 0.001; ΔR*^2^ *= 0.026, ΔF(1, 285) = 8.60, p = 0.004*						
***Model 5. + Mediterranean diet adherence***						
*Constant*	13.612	1.522	10.616 to 16.608	–	8.94	<0.001 **
Age	−0.099	0.038	−0.174 to −0.024	−0.142	−2.60	0.010 *
Sex (female)	0.374	0.540	−0.689 to 1.437	0.040	0.69	0.489
BMI	−0.011	0.047	−0.104 to 0.082	−0.013	−0.23	0.817
Home-cooking frequency (days/week)	−0.121	0.077	−0.273 to 0.031	−0.089	−1.57	0.118
Food-ordering frequency (times/week)	0.076	0.102	−0.125 to 0.277	0.043	0.74	0.459
Cooking Skills score	0.013	0.013	−0.013 to 0.039	0.096	0.97	0.332
Food Skills score	−0.029	0.009	−0.047 to −0.011	−0.325	−3.34	<0.001 **
MEDAS total score	−0.545	0.117	−0.775 to −0.315	−0.251	−4.64	<0.001 **
*R*^2^ *= 0.209; adjusted R*^2^ *= 0.187; F(8, 284) = 9.37, p < 0.001; ΔR*^2^ *= 0.060, ΔF(1, 284) = 21.56, p < 0.001*						
***Model 6. + Digital healthy diet literacy (final model)***						
*Constant*	13.366	1.632	10.154 to 16.578	–	8.19	<0.001 **
Age	−0.101	0.038	−0.176 to −0.026	−0.144	−2.63	0.009 *
Sex (female)	0.405	0.546	−0.670 to 1.480	0.044	0.74	0.459
BMI	−0.012	0.047	−0.105 to 0.081	−0.014	−0.25	0.803
Home-cooking frequency (days/week)	−0.121	0.077	−0.273 to 0.031	−0.090	−1.57	0.117
Food-ordering frequency (times/week)	0.079	0.102	−0.122 to 0.280	0.044	0.77	0.443
Cooking Skills score	0.012	0.013	−0.014 to 0.038	0.093	0.93	0.352
Food Skills score	−0.029	0.009	−0.047 to −0.011	−0.325	−3.33	<0.001 **
MEDAS total score	−0.549	0.118	−0.781 to −0.317	−0.252	−4.65	<0.001 **
e-HDL total score	0.009	0.021	−0.032 to 0.050	0.023	0.42	0.675
*R*^2^ *= 0.209; adjusted R*^2^ *= 0.184; F(9, 283) = 8.33, p < 0.001; ΔR*^2^ *= 0.000, ΔF(1, 283) = 0.18, p = 0.675*						

* *p* < 0.05, ** *p* < 0.001. B = unstandardized coefficient; β = standardized coefficient. BMI: Body mass index, e-HDL: e-Healthy Diet Literacy Scale, MEDAS: Mediterranean Diet Adherence Screener.

## Data Availability

The data presented in this study are available on request from the corresponding author due to privacy restrictions on participant data.
